# Affective Cortical Asymmetry at the Early Developmental Emergence of Emotional Expression

**DOI:** 10.1523/ENEURO.0042-20.2020

**Published:** 2020-08-27

**Authors:** Elaina Bolinger, Hong-Viet V. Ngo, Vanessa Kock, Dirk T. Wassen, Tamara Matuz, Niels Birbaumer, Jan Born, Katharina Zinke

**Affiliations:** 1 Institute of Medical Psychology and Behavioral Neurobiology, University of Tübingen, 72074 Tübingen, Germany; 2 Donders Institute for Brain, Cognition and Behaviour, Radboudumc, 6525 EN Nijmegen, The Netherlands; 3 Wilhelm-Schickard-Institute for Computer Science, University of Tübingen, 72074 Tübingen, Germany; 4 Wyss Center for Bio and Neuroengineering, 1202 Geneva, Switzerland; 5 Werner Reichardt Center for Integrative Neuroscience, University of Tübingen, 72076 Tübingen, Germany

**Keywords:** asymmetry, development, EEG, EKG, emotion

## Abstract

Emotions have an important survival function. Vast amounts of research have demonstrated how affect-related changes in physiology promote survival by effecting short-term and long-term changes in adaptive behavior. However, if emotions truly serve such an inherent function, they should be pervasive across species and be established early in life. Here, using electroencephalographic (EEG) brain activity we sought to characterize core neurophysiological features underlying affective function at the emergence of emotional expression [i.e., at the developmental age when human infants start to show reliable stimulus-elicited emotional states (4–6 months)]. Using an approach that eschews traditional EEG frequency band delineations (like theta, alpha), we demonstrate that negative emotional states induce a strong right hemispheric increase in the prominence of the resonant frequency (∼5–6 Hz) in the infant frontal EEG. Increased rightward asymmetry was strongly correlated with increased heart rate responses to emotionally negative states compared with neutral states. We conclude that functional frontal asymmetry is a key component of emotional processing and suggest that the rightward asymmetry in prominence of the resonant frequency during negative emotional states might reflect functional asymmetry in the central representation of anatomically driven asymmetry in the autonomic nervous system. Our findings indicate that the specific mode hallmarking emotional processing in the frontal cortex is established in parallel with the emergence of stable emotional states very early during development, despite the well known protracted maturation of frontal cortex.

## Significance Statement

Emotions provide an important survival function and are associated with unique brain states that should theoretically be present early in life. In this article, we demonstrate in 4- to 6-month-old infants (i.e., when infants first reliably express emotions) that negative emotions are linked to increased prominence in the resonant EEG frequency of the right compared with the left frontal cortex. The extent of this asymmetry was linked to heart rate reactivity. These results show that emotion induces asymmetric patterns in brain activity at the emergence of emotional expression, possibly laying the foundation for more complex functional asymmetries observed later in life.

## Introduction

Emotions are linked to specific modes of information processing, associated with changes in activity patterns among brain regions like the frontal cortex, amygdala, and hippocampus (LaBar and Cabeza, 2006; Hamann, 2012; Herry and Johansen, 2014). Examining central and autonomic nervous system activity using electroencephalography (EEG) and heart rate, respectively, during emotional expression in infancy provides a unique opportunity to study electrophysiological features of emotion before voluntary control of emotional expression and inhibition are developed, providing insight into both the physiology and functional role of emotions.

In adults, emotional states and emotional processing have been connected to asymmetric power over the frontal cortex in the theta (∼3–7 Hz; Aftanas et al., 2002) and alpha (∼8–15 Hz; Harmon-Jones et al., 2010) frequency bands of the EEG. Depending on the frequency band examined, power increases are sometimes considered inhibitory (i.e., higher alpha is associated with lower “activation”; Jensen and Mazaheri, 2010), and sometimes considered a marker of increased processing (i.e., higher theta is related to enhanced encoding; Lisman and Jensen, 2013). Because frequencies that could be considered as theta or alpha are investigated in this work, all data will be discussed in terms of power rather than “activation.”

The investigation of functional asymmetry in infants is complicated by the developmental change of resonant frequencies in the EEG (i.e., frequency bands that reflect a genuine underlying neural oscillator; Marshall et al., 2002; Saby and Marshall, 2012). Research suggests that infant theta ranges from 2 to 6 Hz (Jones et al., 2015; Michel et al., 2015), whereas infant alpha ranges from 6 to 9 Hz (Hill et al., 2020). While a 6–9 Hz alpha band peak may emerge by the end of the first year, in one study (Xie et al., 2018) only 7% of infants at the age of 6 months exhibited a distinct peak in this range, leading the authors to suggest that infant activity in the neighboring theta band “may be a more reliable neural index of infant cognitive processes in the first few months of life.” In light of these developmental influences interacting in a highly complex manner with frequency and function of EEG oscillators, isolating and analyzing the resonant frequency, as a more data-driven approach to the oscillatory nature of the EEG signal, seems of particular relevance when investigating the EEG in infants.

Although band delineations are a matter of debate, functional asymmetry (i.e., in response to speech processing) emerges early in life (Mai et al., 2014). Observing others express emotion is associated with asymmetric activity in 8-month-old infants (Missana et al., 2014) and 10-month-old infants (Davidson and Fox, 1982). To date, only one study has investigated EEG patterns specifically during infant expression of emotion. In 10-month-old infants (Fox and Davidson, 1988), greater power in a 3–12 Hz band was found in the right hemisphere compared with the left hemisphere during positive emotional expression, while greater power in the left hemisphere was found during expression of negative emotions. Using emotion-induction paradigms (which include data from periods of both clear emotional expression as well as emotional ambiguity), researchers found similar patterns in 6- and 10-month-old infants, demonstrating that those with greater power in the 5/6–9 Hz band in the left hemisphere during negative emotional scenarios also expressed more sadness and/or fear (Buss et al., 2003; Diaz and Bell, 2012).

Here, we investigated EEG and heart rate during the expression of emotions in 4- to 6-month-old infants. To acquire data from negative emotional states (wherein the infant expressed withdrawal behaviors and fussing/crying) and positive states (wherein the infant expressed approach behaviors and smiling), a social interaction paradigm, in which parents elicited infant emotion by acting out naturalistic emotional prompts, was used. Emotionally neutral data were collected from periods wherein infants did not express emotion and no prompts were given. Frontal EEG asymmetry of the resonant frequency (i.e., the most prominent peak in the 3–15 Hz range) was analyzed. At this early stage of development (before the development of behavioral inhibition), we anticipated that frontal EEG asymmetry reflects an anatomically driven difference in the central representation of sympathetic nervous system activity, with greater sympathetic engagement (i.e., during negative states) leading to rightward EEG asymmetry (Craig, 2005). We expected the degree of this asymmetry would be related to heart rate increases during negative states, as sympathetic increases in heart rate activity have been related to increase activity in the frontal right cortex (Oppenheimer et al., 1992). Conversely, positive emotional states that are more closely related to increased activity of the parasympathetic nervous system (McCraty et al., 1995) were expected to induce a leftward asymmetry.

## Materials and Methods

### Participants

Thirty infant–parent dyads were recruited with the intention to retain a minimum of *n* = 24 infants for final analyses. G*Power was used to calculate the *n* = 24 sample size based on a standard power value of 0.80, an α error probability of 0.05, and an effect size of 0.35 for a 2 × 3 repeated-measures ANOVA, reflecting the focal 2 × 3 (hemisphere × emotion) design of this study. All infants were born at full term and were healthy according to parental report. Infants were excluded if they had received a diagnosis of any chronic medical condition (e.g., epilepsy, cardiac arrhythmia) and/or were taking medication on a regular basis. In addition, parents were asked to postpone experimental sessions if their infant exhibited cold-like symptoms such as fever, coughing, runny nose, or diarrhea. Dyads were allowed to participate only if the infant was still in a prelocomotor stage, as the ability to crawl influences the types of stimuli that elicit emotion (Fox and Henderson, 2007), as well as the pattern and occurrence rate of expression behaviors (Scarr and Salapatek, 1970; Oster et al., 1992; Leppänen and Nelson, 2009). Data from two participants were excluded because of technical problems, and another three participants were excluded because of insufficient data. The final sample consisted of 25 infants (*n* = 12 females) who were between the ages of 17.7 and 31.7 weeks (mean ± SD: 22.9 ± 3.7 weeks). Infant Behavior Questionnaire (IBQ) scores for Surgency (4.43 ± 0.60), Negative Affect (3.87 ± 1.18), and Effortful Control (4.97 ± 0.69) demonstrated that infants were emotionally healthy and comparable to infants of similar age (Putnam et al., 2014). For the final sample, parents consisted of 24 mothers and 1 father, and all were the primary caregivers of the infant. According to self-report in the initial interview, all parents had not received a diagnosis of postpartum depression after the birth of the participating infant or other children. Parents received 60€ for participation. The study was approved by the local ethical commission, and all parents gave written informed consent for participation.

### Emotion elicitation paradigm

The emotion elicitation paradigm was designed based on previously developed interaction experiments (Fox and Davidson, 1987; Dawson et al., 1992; Trainor et al., 2000; Nakata and Trehub, 2004) and with the goal of maximizing the infants’ emotional expressions. Parents were asked to serve as eliciting agents as it was expected that parents (1) had the experience to know how to best elicit emotions from their infants, and (2) parents could dynamically adapt their behavior to optimize the infants’ emotional response. Five prompts for the emotional interactions were used to elicit positive and negative emotions. The positive prompts were as follows: (1) love: express love to your infant; (2) peek-a-boo: play a game of peek-a-boo with your infant; and (3) sing: sing to your infant. The negative prompts were as follows: (4) rash: pretend your infant has a rash on his/her face; and (5) electrical outlet: pretend your child has crawled to an electrical outlet. A sixth prompt (jack-in-the-box, wherein the parent played with and demonstrated a jack-in-the-box toy to the infant) was excluded from analyses because it did not reliably induce one specific emotional valence as determined by behavioral criteria.

Emotionally neutral behavior was collected during periods when no emotional prompts were given, the infant expressed no overt emotion (according to scoring criteria), and the experimenter and parents spoke to each other in adult-directed speech.

### Experimental procedure

Descriptions of the emotional prompts were sent to parents in advance to give them time to envision how they would enact each scenario. Upon arrival at the laboratory, the infant was given time to adapt to the environment and experimenters while the parent filled out the Very Short Version of the Infant Behavior Questionnaire (Putnam and Rothbart, 2006) and was shown example videos of other parents acting out the emotional prompts. EEG, electrooculography (EOG), and electrocardiography (EKG) electrodes were applied while the parent played with the infant. A video camera was positioned at an angle that captured a frontal view of the infant’s face and body, as well as a profile view of the parent. The emotion elicitation paradigm was then initiated. One trial consisted of a parent’s attempt to act out a prompt and the infant’s response. Multiple trials were collected for each prompt type. The experimenter told the parent which prompt to enact and manually inserted start and end markers into the physiological data for each trial. Prompts were executed in a semirandom order in which consecutive repetition of the same prompt was avoided. Trials were separated by breaks lasting between 1 and 30 min, flexibly taking into account the infant’s needs (e.g., feedings and diaper changes) as well as giving the infant time to return to a calm state if they became emotionally aroused for an extended period of time. EEG quality was checked before initiating a new trial to ensure the cap remained in the correct position, and that electrode impedances were <10 kΩ. Sessions were limited to 2 h, and parents were allowed to initiate breaks or terminate the session at any time. The number of negative trials and the length of negative trials were restricted according to general guidelines for infants’ daily limits for negative events (i.e., not exceeding the number of negative events an infant would experience in a normal day; Fox and Henderson, 2007). To acquire a substantial number of trials, a second, largely identical elicitation session was recorded within 1–5 d of the first session (thus, each infant-parent dyad participated for ∼4 h in total).

### Scoring of emotional behavior

The video-recorded infant behavior was scored offline by two independent raters. Scoring of infants’ emotional behavior during each trial was based on primary and secondary criteria (Table 1), which were adapted from multiple sources to include facial (Sullivan and Lewis, 2003), vocal, and bodily (Goldsmith and Rothbart, 1996) expressions of emotion in prelocomotor infants. Raters first determined whether or not the trial was successful at eliciting the target emotional state (i.e., positive emotion elicited by prompts 1–3, negative emotion elicited by prompts 4 and 5) by determining whether primary criteria (behaviors that clearly express emotional state; Table 1) were expressed at any point during the parent’s attempt. Videos included both the parent and the infant, often interacting and expressing similar emotions synchronously; therefore, in this study it was not possible to keep the raters blinded to the target emotional state. Positive trials were deemed successful if the infant smiled or laughed and made eye contact with the parent, and negative trials were deemed successful if the infant fussed or cried. An inter-rater reliability analysis showed that raters largely agreed as to whether or not the infants expressed the targeted emotions during trials (Cohen’s κ = 0.71, *p *<* *0.001) see Table 2 for overview of emotion elicitation success. To extract physiological data corresponding to emotional states, raters inserted markers at the beginning and ending of an infant’s emotional response. An emotional response could consist of multiple behaviors that met primary and secondary criteria (Table 1). For example, the onset marker of a positive response might be when an infant coos (secondary). The infant might then reach for the parent (secondary), smile (primary), and start giggling (primary). The offset marker would then be placed at the end of the giggling, once the emotional response is complete and no more emotional expression could be observed. A negative response might consist of the infant pushing the parent away (secondary), arching their back (secondary), and then crying (primary). Emotionally neutral states were defined as time periods when the parent and experimenter did not interact with the infant and the infant expressed no overt emotion (i.e., raters scored neutral states to ensure none of the behavior listed as primary criteria for negative and positive states were observed during these periods; Table 1). For an emotional response to be used in further analyses, it was necessary that the infant’s emotional expression contained the primary criteria for the respective valence at least once within the emotional response period.

**Table 1:** Primary criteria (behaviors that clearly express emotional state) and secondary criteria (behaviors that are associated with emotional state but require contextual information to be interpreted) used in behavioral scoring

	Neutral state	Positive state	Negative state
Primary criteria	No overt emotion	Smiling + eye contact, laughter/giggling	Fussing/crying
Secondary criteria	Adults talking	Cooing/babbling, approach (reaching), waving arms/legs	Pushing away, avoidance (arching, looking away)

**Table 2:** Mean (±SD) number of parent attempts and success rate according to emotional prompt type for all *n *=* *25 infant–parent dyads

Prompt	Target emotional state	Parent attempt #	Success rate
1. Love	Positive	9.08 ± 4.11	0.38 ± 0.30
2. Sing	Positive	7.24 ± 3.63	0.26 ± 0.27
3. Peek-A-Boo	Positive	7.60 ± 1.96	0.52 ± 0.31
4. Rash	Negative	4.24 ± 1.51	0.86 ± 0.16
5. Electrical outlet	Negative	3.20 ± 1.44	0.58 ± 0.40

### Recordings and data analysis

EEG, EOG, EKG, and video were collected using a portable amplifier (SOMNOscreen plus EEG 32) and DOMINO version 2.6.1 recording software, Somnomedics). EEG (F3, Fz, F4, C3, C4, P3, Pz, and P4) was recorded using stretch caps with soft Ag/Cl electrodes (EASYCAP). Eye movements were recorded via a bipolar EOG with diagonally placed electrodes (one electrode below the left eye and the other at Fp2). The EKG electrode was attached below the left breast. Physiologic signals (EEG and EKG) were collected using Cz and Fp1 as the reference and ground electrodes, respectively. Signals were sampled at 256 Hz, and impedances were kept under 10 kΩ. Video was recorded synchronously with the physiological signals via an Axis M1034 camera (Axis Communications). After offline behavioral scoring (using the DOMINO software), which was done to mark recording epochs with consistent emotional expressions (see Scoring of emotional behavior), electrophysiological signals were exported to the Brain Vision Analyzer 2.0 Software (Brain Products) for data cleaning and preprocessing. These values were further analyzed with MATLAB version 2014b (MathWorks).

### EEG power spectral density analysis

EEG corresponding to periods of emotional expression (i.e., the scored and extracted neutral, positive, and negative states) during the different prompts was bandpass filtered between 1 and 30 Hz using an IIR Butterworth zero lag filter (fourth order) with a 50 Hz notch filter. Data were then segmented into 2 s epochs with 1 s overlap. Automatic artifact rejection was applied to these epochs, such that segments containing (1) a voltage step above 75 µV/ms, (2) a voltage difference of 100 µV within 100 ms, (3) a voltage exceeding ± 100 µV, or (4) a period with signal fluctuations within ±0.5 µV persisting for >100 ms on any channel (F3, Fz, F4, C3, C4, P3, Pz, P4) were removed from further analysis. After artifact rejection, on average 133.8 s of neutral data, 147.4 s of positive data, and 32.2 s of negative data were available per infant. Finally, all channels were rereferenced to an average reference.

Power spectral density (PSD) was then calculated for each artifact-free epoch using irregular-resampling auto-spectral analysis (IRASA; Wen and Liu, 2016), a procedure that has proven to be effective at isolating oscillatory signals from background noise. These values were normalized according to electrode and condition by dividing each 0.5 Hz bin by the area under the curve between 1 and 30 Hz. Spectra were then averaged according to emotional state (positive: prompts 1–3; negative: prompts 4 and 5) for each participant.

We then investigated the “resonant frequency” defined here as the peak between 3 and 15 Hz with the greatest “prominence” in an infant’s EEG spectrum for each emotional state (neutral, positive, and negative; Table 3). Unlike absolute peak magnitude, prominence measures the magnitude of the peak relative to the neighboring topography of the frequency spectrum, thus focusing the analysis on oscillations that reflect a strong resonant frequency in the underlying neural activity. The resonant frequency was characterized in terms of the frequency bin at which it occurred, the prominence value at this frequency (i.e., peak magnitude relative to the highest trough between said peak and neighboring peaks), and the PSD value at this frequency (Table 3). This was performed for F3 and F4 separately, as well as P3 and P4 (C3 and C4 were not analyzed). Asymmetry was always calculated as F4–F3; therefore, positive values represent a rightward asymmetry and negative values represent a leftward asymmetry.

**Table 3:** Electrophysiological data according to emotional state

	Neutral		Positive		Negative	
	F3	F4	F3	F4	F3	F4
Resonant frequency (Hz), mean ± SD	6.14 ± 2.23	5.82 ± 2.61	5.80 ± 2.02	6.30 ± 2.32	5.42 ± 1.61	5.12 ± 1.60
Resonant frequency (Hz), range	3–12.5	3.5–14	3–11.5	4–12.5	3–9	3–10.5
Resonant frequency prominence (μV^2^/Hz), mean ± SD	0.063 ± 0.060	0.082 ± 0.064	0.061 ± 0.072	0.045 ± 0.047	0.074 ± 0.062	.107 ± 0.093
Resonant frequency PSD (μV^2^/Hz), mean ± SD	0.070 ± 0.066	0.084 ± 0.062	0.073 ± 0.070	0.058 ± 0.049	0.088 ± 0.074	0.112 ± 0.087
Heart rate (beats/min), mean ± SD	141.11 ± 12.08		136.44 ± 9.56		155.62 ± 12.42	

The resonant frequency was defined as the most prominent peak in the 3–15 Hz range.

### Heart rate analysis

To investigate the influence of emotion on heart rate, 10 s artifact-free EKG segments were extracted from the emotional state data. QRS complexes were detected using the Brain Vision Analyzer 2.0 Software, and the number of R peaks in each epoch was multiplied by six to obtain heart rate in beats per minute. Heart rate epochs were then pooled and averaged by emotional state for each participant. For a participant to be included in the analyses, a minimum of one 10 s artifact-free epoch was required for every emotional state. One participant had to be excluded because of artifacts in the EKG data.

### Statistical approach

Modulation of asymmetric cortical engagement by emotional state was investigated using repeated-measures ANOVA with the factors hemisphere (F3 vs F4) and emotion (neutral, positive, negative). This was applied to all characteristics (i.e., frequency, prominence, power) of the resonant frequency. Greenhouse–Geisser correction was used for *p* values when appropriate (with degrees of freedom left unchanged for the sake of clarity). Paired *t* tests were used to follow up significant effects. The α value was set at 0.05 for these tests. To ascertain whether influences were restricted to frontal sites, the factor region (frontal vs parietal) was added to the hemisphere × emotion ANOVA, with a subsequent hemisphere × emotion ANOVA applied to parietal electrodes (P3 vs P4).

Affective modulation of heart rate was investigated using a repeated-measures ANOVA with the factor emotion. Pearson’s correlations were used to explore possible relationships between valence-specific emotional reactivity (e.g., difference between negative and neutral state) in EEG asymmetry and heart rate, as well as potential relationships between neutral (trait) EEG asymmetry and IBQ emotional response variables.

## Results

### Negative affect induces frontal rightward asymmetry in resonant frequency

**Figure 1.**
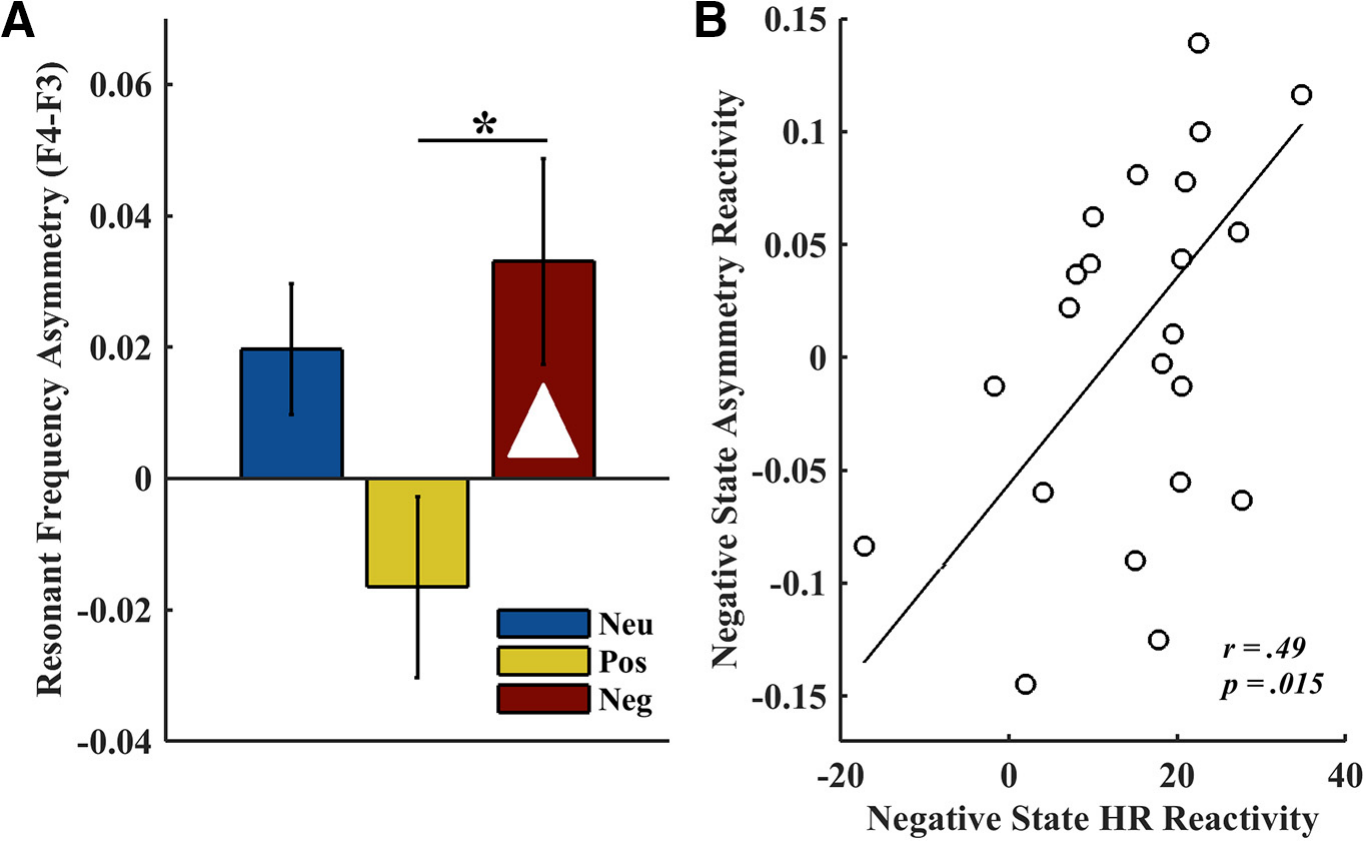
A negative affective state induces rightward asymmetry, which is related to emotional heart rate reactivity. ***A***, Affective state influenced the asymmetry (F4–F3, mean ±SEM) in the prominence of the most resonant frequency in the infant frontal EEG (∼5–6 Hz). Positive (yellow) emotional states were associated with relatively greater prominence on the left, compared with negative (red) and neutral (blue) states. Robust asymmetry (significantly differing from zero, represented as a white triangle) was only induced by negative affective states. *p < 0.05. ***B***, Greater asymmetry reactivity predicted greater heart rate reactivity during negative states (reactivity measured as the difference between negative and neutral states).

Emotion modulated the prominence of the resonant frequency at the frontal electrode sites (F3, F4) in a hemisphere-dependent manner (hemisphere × emotion interaction: *F*_(2,48)_ = 3.66, *η*_p_^2^ = 0.13, *p *=* *0.033; main effect hemisphere: *F*_(1,24)_ = 2.50, *η*_p_^2^ = 0.09, *p *=* *0.13; main effect emotion: *F*_(2,48)_ = 3.63, *η*_p_^2^ = 0.13, *p *=* *0.034). In light of these hemispheric differences, we calculated an asymmetry score (F4–F3) between prominence values to follow up this significant interaction. Negative affect induced a rightward asymmetry in the prominence of the resonant frequency (∼5–6 Hz; Table 3), which was in contrast to the leftward asymmetry observed during positive emotional states (*t*_(24)_ = −2.98, *p *=* *0.007, *d *=* *0.59; Fig. 1*A*). Neutral states did not significantly differ from negative states (*t*_(24)_ = −0.63, *p *=* *0.54, *d *=* *0.13) or positive states (but note the trend: *t*_(24)_ = 1.94, *p *=* *0.064, *d *=* *0.39). The rightward asymmetry during negative states was robust because the F4–F3 asymmetry score significantly differed from zero (*t*_(24)_ = 2.11, *p *=* *0.045, *d *=* *0.42). Asymmetry scores during the other states did not statistically differ from zero (neutral: *t*_(24)_ = 1.98, *p *=* *0.06, *d *=* *0.40; positive: *t*_(24)_ = −1.20, *p *=* *0.24, *d *=* *0.25).

An analysis including parietal electrodes (P3, P4) confirmed that this effect was localized frontally, as follows: region (frontal vs parietal) was found to influence how emotional state modulated prominence across hemispheres (hemisphere × region × emotion interaction: *F*_(2,48)_ = 4.23, *η*_p_^2^ = 0.15, *p *=* *0.033). An ANOVA investigating hemisphere and emotion at parietal recordings did not indicate any significant change in the asymmetry of prominence of the resonant frequency that depended on the experimentally induced emotion (parietal hemisphere × emotion interaction: *F*_(2,48)_ = 2.50, *η*_p_^2^ = 0.09, *p *=* *0.11). Overall, these results therefore suggest that the emotional modulation of resonant frequency prominence is frontally localized.

Because of the relatively low amount of data in the negative compared with positive conditions, we correlated the difference in available epochs between positive and negative conditions with the difference in frontal asymmetry between positive and negative conditions. Opposite to our target finding, the correlation coefficient pointed toward greater differences in available epochs to be associated with greater prominence on the right during positive states and on the left during negative states, respectively, and was not significant (*r *=* *0.32, *p *=* *0.13). Altogether, these data exclude a substantial contamination of our target results by differences in the available amount data for the emotional conditions.

### Investigation of asymmetry in resonant frequency PSD and frequency

**Figure 2.**
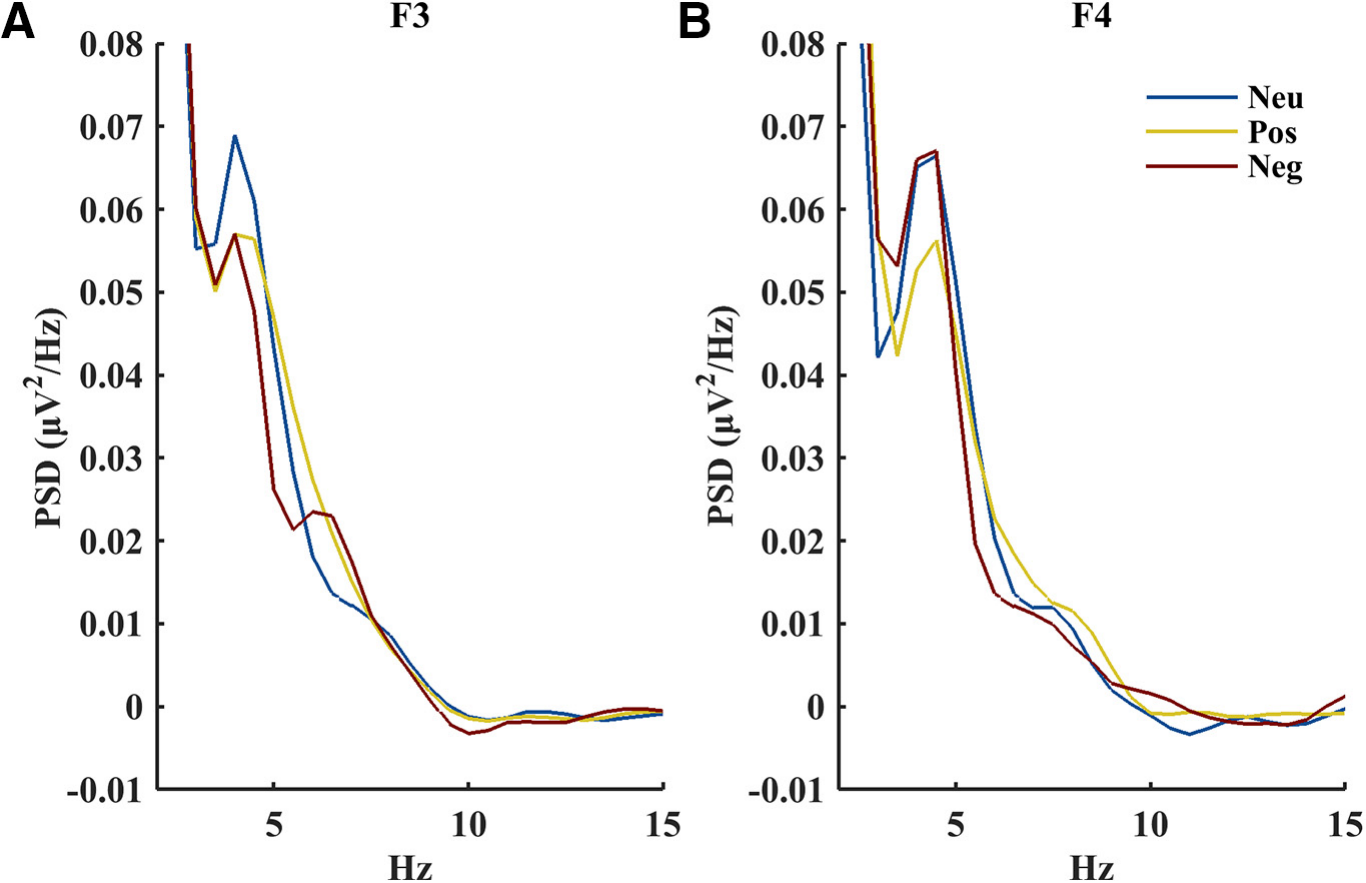
Power spectral density according to infant emotional state (neutral: blue, positive: yellow, negative: red) at F3 (***A***) and F4 (***B***). PSD values for each 0.5Hz bin were normalized by dividing by the area under the curve within 1–30 Hz.

Descriptively, a similar influence of emotion on resonant frequency asymmetry over the frontal cortex (F3, F4) could also be observed in the resonant frequency PSD value, though the corresponding hemisphere × emotion interaction did not reach significance (*F*_(2,48)_ = 2.75, η_p_^2^ = 0.10 *p *=* *0.074; main effect of hemisphere: *F*_(1,24)_ = 0.89, *η*_p_^2^ = 0.04, *p *=* *0.36; main effect emotion: *F*_(2,48)_ = 2.85, *η*_p_^2^ = 0.11, *p *=* *0.068). Power spectra at F3 and F4 can be found in Figure 2.

For the frequency of the resonant peak, a trend effect of emotion was found, wherein negative states appeared to be associated with lower frequencies (main effect of emotion: *F*_(2,48)_ = 2.59, *η*_p_^2^ = 0.10, *p *=* *0.085). No hemispheric influences were observed (main effect: *F*_(1,24)_ = 0.037, *η*_p_^2^ = 0.002 *p *=* *0.85; hemisphere × emotion interaction: *F*_(2,48)_ = 0.74, *η*_p_^2^ = 0.03, *p *=* *0.48), indicating that the resonant frequency of interest did not differ according to hemisphere. Given the trend toward lower frequencies during negative states, we additionally explored whether the frequency of the resonant peak (here taken as an average between F3 and F4, in light of no main effect of hemisphere) was related to asymmetry. Resonant peak frequency and asymmetry were not related during neutral (*r* = −0.092, *p *=* *0.66) or positive (*r *=* *0.221, *p *=* *0.29) states. A trend relationship (*r* = −0.36, *p *=* *0.077), wherein lower peak frequencies were related with greater rightward prominence during negative states was observed. Together, these findings suggest that rightward asymmetry during negative states recruits a slower resonant frequency.

### Negative affect-induced frontal asymmetry is associated with stronger heart rate response

Negative emotional states were associated with higher heart rate than positive (*t*_(23)_ = −8.79, *p *<* *0.001, *d *=* *1.80) and neutral states (*t*_(23)_ = −6.21, *p *<* *0.001, *d *=* *1.27; main effect of emotion: *F*_(2,46)_ = 38.04, *η*_p_^2^ = 0.62, *p *<* *0.001). The difference between positive and neutral states did not reach significance, though heart rate was descriptively lower during positive emotional states (Table 3; *t*_(23)_ = 1.98, *p *=* *0.06, *d *=* *0.40). To investigate whether EEG asymmetry and heart rate were related, reactivity scores were calculated for both frontal asymmetry in the prominence of the resonant frequency and heart rate, whereby reactivity was expressed as the difference in the measure (EEG, heart rate) between the emotional state of interest (negative, positive) and the neutral state. These analyses revealed that greater frontal asymmetry reactivity induced by negative emotional states (i.e., a larger rightward asymmetry during negative compared with neutral states) was associated with a stronger heart rate increase (*r *=* *0.49, *p *=* *0.015; Fig. 1*B*). The same association was not found for positive emotional reactivity (*r *=* *0.34, *p *=* *0.10).

Because many studies have suggested that baseline asymmetry (i.e., in resting conditions) is predictive of general emotional reactivity (Jones et al., 1997; Buss et al., 2003; Diaz and Bell, 2012; Gartstein et al., 2014; Licata et al., 2015; Wen et al., 2017), we additionally correlated asymmetry in the resonant frequency during neutral states (i.e., a resting condition) with both negative and positive heart reactivity and parent-reported IBQ measures (Surgency, Negative Affect, Effortful Control). Resonant frequency asymmetry during neutral states was not related to heart reactivity or IBQ measures (*p* values >0.28).

### Exploration of age and resonant frequency

Because the frequency of the resonant frequency is expected to increase over the first year of life (Marshall et al., 2002), we explored whether the main measures of this study (resonant frequency, frontal asymmetry in resonant frequency, asymmetry reactivity, and heart rate reactivity) were associated with age. Age was not related to the frequency of the resonant frequency in frontal electrodes (correlation based on F3/F4 mean resonant frequency because no hemispheric differences were found; neutral: *r* = −0.061, *p *=* *0.77; positive: *r* = −0.095, *p *=* *0.65; negative: *r* = −0.034, *p *=* *0.87), frontal prominence asymmetry (neutral: *r* = −0.37, *p *=* *0.073; positive: *r *=* *0.112, *p *=* *0.57; negative: *r* = −0.004, *p *=* *0.98), asymmetry reactivity (negative: *r *=* *0.17, *p *=* *0.42; positive: *r *=* *0.28, *p *=* *0.17), or heart rate reactivity (positive: *r = *0.10, *p *=* *0.63; negative: *r* = −0.19, *p *=* *0.63). It therefore seems unlikely that age influenced the main findings of this research.

### Trial type control analyses

To ensure that asymmetry differences were not driven by a specific trial type (especially given that positive trials contained both infant-directed talking and infant-directed singing, which have been shown to elicit differing behavioral patterns in infants; Arias and Peña, 2016), resonant frequency analyses were repeated separately for each emotional state (positive: Love, Sing, Peek-a-Boo; negative: Rash, Electrical Outlet). For positive states, 10 infants had data available for each type of trial (Love, Sing, Peek-a-Boo). Trial type did not influence the frequency of the resonant frequency or the prominence of that peak (hemisphere × trial type and main effect, *p* values >0.11). Playing Peek-A-Boo was associated with lower resonant frequency PSD (main effect of trial type: *F*_(2,18)_ = 3.97, *η*_p_^2^ = 0.31, *p *=* *0.037, observed power = 0.64; hemisphere × trial type interaction and main effect, *p* values* >*0.16) and generally fewer peaks (aside from the main resonant peak, main effect of trial type: *F*_(2,18)_ = 8.53, *η*_p_^2^ = 0.49, *p *=* *0.002, observed power = 0.93; hemisphere × trial type interaction and main effect, *p* values* >*0.083). For negative states, 13 infants had data for each type of trial (Rash, Electrical Outlet). No aspects of the resonant frequency (frequency, prominence, power) or the number of peaks in general were related to trial type (hemisphere × trial type and main effect, *p* s >0.10). While the responses to the different trial types were characterized by a rather large variability, overall the control analyses did not provide evidence that a single trial type of positive or negative emotional state was likely to drive the main results of this study.

### Investigation of infant EEG 6–9 Hz alpha

Because previous work has generally investigated the influence of emotion within a 6–9 Hz band in infants, we additionally analyzed asymmetry (F4–F3) in the average power in the oscillatory signal in this range. Note that a logarithmic transformation, as is often performed on power data generated by FFT procedures, was not used here because the IRASA procedure used in this study removes the 1/f noise that generally necessitates logarithmic transformation before statistical analysis. Emotion was not found to influence asymmetry in the 6–9 Hz band of the oscillatory signal (*F*_(2,48)_ = 0.69, *η*_p_^2^ = 0.03, *p *=* *0.45).

We additionally investigated asymmetry in the mixed IRASA output (i.e., before 1/f noise is removed), which corresponds to the standard approach to asymmetry analyses taken in previous studies (Coan and Allen, 2003; Smith et al., 2017). Power was again averaged in the 6–9 Hz range, but this time a logarithmic transformation was applied before calculating the asymmetry score. Here, an influence of emotion was found (*F*_(2,48)_ = 4.51, *η*_p_^2^ = 0.16, *p *=* *0.03), wherein negative states were associated with more power in the left hemisphere (−0.044 ± 0.18) compared with neutral states (*t*_(24)_ =2.49, *p *=* *0.02, *d *=* *0.48), and neutral states were associated with more power in the right hemisphere (0.059 ± 0.12) compared with positive states (0.00 ± 0.10, *t*_(24)_ = 2.85, *p *= 0.009, *d *=* *0.60). Note, reactivity in this 6–9 Hz asymmetry (difference between negative and neutral asymmetry) was not correlated with heart rate reactivity during negative states (*r* = −0.22, *p *=* *0.31), nor was reactivity in asymmetry (difference between positive and neutral asymmetry) related to heart rate reactivity during positive states (*r *=* *0.11, *p *=* *0.61). It therefore does not seem that asymmetry in the infant alpha band (6–9 Hz) derived from the mixed power spectrum (oscillatory plus fractal signals combined) is related to autonomic arousal, as was observed for resonant frequency prominence in this study.

## Discussion

Negative emotional states induced a rightward asymmetry in the most prominent oscillation (∼5–6 Hz) in the 4- to 6-month-old infant frontal EEG, which could be differentiated from a leftward asymmetry seen during positive emotional states, but not from asymmetry during neutral states. Frontal rightward asymmetry induced by negative states was strongly associated with increases in heart rate during negative states, reflecting a pattern that has also been observed in adults (Balconi et al., 2015).

This study is the first to explore neurophysiological signatures of emotional expression at the developmental onset of robust stimulus-elicited affective expression (though note that a number of studies have investigated EEG during emotion induction procedures; Buss et al., 2003; as well as the observation of expression by others, e.g., Davidson and Fox, 1982). While the elicitation of negative emotion is already possible at birth, linking an external stimulus to positive emotion (a prerequisite for the straightforward experimental manipulation of emotional state) only becomes possible around 3–4 months (e.g. Messinger and Fogel, 2007). Our results are therefore uniquely positioned to provide insight into the basis of affective processing and in particular, asymmetric affective engagement of the frontal cortex. The underlying factor driving frontal asymmetries in adult populations has thus far evaded clear delineation. Asymmetry in power has been found to be related to functional differences in autonomic nervous system engagement (rightward asymmetry during sympathetic activation; Craig, 2005). Alternatively, it has been connected to the valence of the emotional state (rightward power asymmetry during positive states; Davidson and Fox, 1982), to approach motivation (rightward power increase in scenarios with approach motivation, like happiness or anger; Coan and Allen, 2004), and supervisory control (rightward power increase being related to decreased emotion regulation effort; Gable et al., 2015). Our finding of a strong link between right frontal asymmetry and heart rate reactivity induced by negative states lends support to the theory that affective asymmetry in the frontal cortex might be driven by a functional asymmetry in the central representation of the autonomic nervous system, which arises from both efferent and afferent anatomic asymmetries in the peripheral nervous system (Craig, 2005).

At first glance, our results appear to be at odds with approach motivation theory (Coan and Allen, 2004), which posits that behaviors associated with the motivation to approach a scenario (e.g., happiness, anger) are associated with increases in power in the right hemisphere. In this study, negative and positive emotional states were associated with withdrawal and approach behaviors, respectively, yet when considering asymmetry in the resonant frequency, we found a relative rightward asymmetry during negative states—the precise opposite of what has been found in previous infant studies (i.e., leftward asymmetry during negative states; Fox and Davidson, 1988; Buss et al., 2003; Diaz and Bell, 2012). Notably, however, our results reflect previous work when asymmetry in the fixed 6–9 Hz band (infant alpha) of the mixed (oscillatory and fractal components combined) EEG signal was considered instead of the resonant frequency in the 3–15 Hz range of the oscillatory signal. This might suggest that asymmetry during negative states varies according to frequency, with theta-like frequencies increasing in prominence on the right, and alpha-like frequencies increasing in prominence on the left. In this view, the asymmetry observed in the 6–9 Hz range is a precursor to alpha asymmetry, which modulates approach/withdrawal later in life. Nevertheless, the 5–6 Hz asymmetry in the opposite direction (which was captured in our analysis of the resonant frequency) seems to be more clearly connected to emotional processing in this age group, in that it reflects the strongest neural oscillator at this developmental stage and rightward asymmetry in this range scaled with autonomic (heart rate) response during negative states. One might argue that a 5–6 Hz band reflects a slow alpha-like activity, which would theoretically mature with time into a faster 6–9 Hz infant alpha. We did not find evidence that this resonant frequency changes within the age range considered (4–6 months), and closer inspection of Figure 2 and individual spectra suggests there are two distinct “theta” and “alpha” bumps in the infant EEG. It seems more likely that this 5–6 Hz resonant frequency is therefore a theta oscillator, as theta power has been repeatedly linked to autonomic activation in adults (Tang et al., 2009; Liou et al., 2014). For example, coupling between autonomic heart activity and central EEG oscillations during emotional arousal has been observed in the theta band (Valenza et al., 2016). From a developmental perspective, it could be argued that the theta network, which spans limbic and prefrontal regions and is involved in the encoding of salient information (Lisman and Jensen, 2013), might emerge earlier and before an inhibitory network such as alpha. In combination with previous findings, our results prompt a novel conceptual view in that negative emotional arousal might first and foremost modulate theta networks and related functions like attention/information encoding (Fries, 2015) rather than alpha and connected functions of behavioral inhibition.

While resting asymmetry has often been linked to emotional predisposition in infants and has been found as early as 3 months of age (Jones et al., 1997; LoBue et al., 2011; Diaz and Bell, 2012; Gartstein et al., 2014; Gabard-Durnam et al., 2015; Licata et al., 2015), we did not find any relationships between resonant frequency asymmetry during neutral states and heart rate reactivity or parent-evaluated emotional trait measures. Although infant-specific patterns in emotional (Brooker et al., 2017) and resting (Jones et al., 1997) asymmetry appear to be rather stable over the first years of life, it is possible that the relationship between resting-state asymmetry and emotional predisposition develops in an experience-dependent manner. In this view, asymmetric emotion-induced activity in the immature frontal cortex may underlie hemispheric differences in the development of the frontal lobe, eventually leading to emotional predispositions. Future studies should therefore investigate how the patterns observed here evolve over the first years of life and how they relate to the development of emotional control.

We conclude that positive and negative emotions are connected to specific modes of brain activity already during early infancy. Rightward asymmetry in the resonant EEG frequency (∼5–6 Hz) over frontal cortex during negative emotional states is related to distinct increases in heart rate during negative states, suggesting that autonomic and central nervous coupling is a fundamental component of emotional processing. Future research should explore how these patterns relate to learning and specifically the gain in capacities of emotional control (Seehagen and Herbert, 2012; Seehagen et al., 2015) and behavioral inhibition.

## Synthesis

Reviewing Editor: Cheryl Sisk, Michigan State University Decisions are customarily a result of the Reviewing Editor and the peer reviewers coming together and discussing their recommendations until a consensus is reached. When revisions are invited, a fact-based synthesis statement explaining their decision and outlining what is needed to prepare a revision will be listed below. The following reviewer(s) agreed to reveal their identity: Sarah Jessen.

All reviewer concerns have been adequately addressed.
